# TCF4 Is a Molecular Target of Resveratrol in the Prevention of Colorectal Cancer

**DOI:** 10.3390/ijms160510411

**Published:** 2015-05-07

**Authors:** Jin Boo Jeong, Jihye Lee, Seong-Ho Lee

**Affiliations:** Department of Nutrition and Food Science, College of Agriculture and Natural Resources, University of Maryland, College Park, MD 20742, USA; E-Mails: jjb8376@gmail.com (J.B.J.); jlee1232@umd.edu (J.L.)

**Keywords:** resveratrol, phytochemicals, TCF4, colon cancer

## Abstract

The Wnt/β-catenin pathway plays an essential role in the tumorigenesis of colorectal cancer. T-cell factor-4 (TCF4) is a member of the TCF/LEF (lymphoid enhancer factor) family of transcription factors, and dysregulation of β-catenin is decisive for the initiation and progression of colorectal cancer. However, the role of TCF4 in the transcriptional regulation of its target gene remained poorly understood. Resveratrol is a dietary phytoalexin and present in many plants, including grape skin, nuts and fruits. Although resveratrol has been widely implicated in anti-tumorigenic and pro-apoptotic properties in several cancer models, the underlying cellular mechanisms are only partially understood. The current study was performed to elucidate the molecular mechanism of the anti-cancer activity of resveratrol in human colorectal cancer cells. The treatment of resveratrol and other phytochemicals decreased the expression of TCF4. Resveratrol decreases cellular accumulation of exogenously-introduced TCF4 protein, but did not change the TCF4 transcription. The inhibition of proteasomal degradation using MG132 (carbobenzoxy-Leu-Leu-leucinal) and lactacystin ameliorates resveratrol-stimulated down-regulation of TCF4. The half-life of TCF4 was decreased in the cells exposed to resveratrol. Resveratrol increased phosphorylation of TCF4 at serine/threonine residues through ERK (extracellular signal-regulated kinases) and p38-dependent pathways. The TCF4 knockdown decreased TCF/β-catenin-mediated transcriptional activity and sensitized resveratrol-induced apoptosis. The current study provides a new mechanistic link between resveratrol and TCF4 down-regulation and significant benefits for further preclinical and clinical practice.

## 1. Introduction

Colorectal cancer is the third most common malignancy in the U.S. and worldwide [1,2]. The development of colorectal cancer is a multistep process accompanied by adenomatous polyps, acquiring a series of somatic mutations and aberrant gene expression [3,4]. The aberrant Wnt signaling pathway plays a major role in development of the early stage of colon cancer [5]. Wnt signaling regulates β-catenin stabilization, which accumulates in the cytoplasm and binds to T-cell factor 4 (TCF4) in colon cancer [6]. The β-catenin/TCF4 complex is localized into the nucleus, where it leads to up-regulation of downstream targets in colon cancer and is implicated in colorectal tumorigenesis [7,8]. Therefore, the interruption of the β-catenin/TCF4 complex could be a potential target in the treatment of colon cancer. Although the formation of a β-catenin and TCF4 complex in the nucleus is well known as a prerequisite for the transcription of the Wnt target gene, TCF4 alone may play a more significant role in tumorigenesis. In fact, compared to β-catenin knockdown, TCF4 knockdown shows better efficacy to induce growth arrest and apoptosis in human colorectal cancer cells [9]. Despite its significant role in transcriptional activation and repression, the role of TCF4 in cancer prevention has remained poorly understood.

The ineffectiveness of the therapeutic approach against colorectal cancer motivates us to consider complementary and alternative medicines moving forward. In alternative medicine, some phytochemicals, widely distributed in vegetables and fruits, are used as a supplement to prevent metabolic syndrome, inflammation and tumorigenesis [10,11,12,13]. Chemoprevention using dietary components is an appropriate and promising strategy. A number of epidemiological studies reported that there is strong inverse relationship between the consumption of vegetables and colorectal cancer [14]. A constant intake of phytochemical-containing plants is beneficial to furnish a defensive mechanism against cancer. A lot of studies have demonstrated that the β-catenin/TCF-dependent signaling pathway is a molecular target for the chemoprevention of phytochemicals [15], and we reported that capsaicin suppressed the expression and protein interaction of β-catenin and TCF4 in human colorectal cancer cells [16].

Resveratrol is a natural polyphenolic phytochemical abundant in grapes, berries and peanuts [17] and possesses a variety of bioactivity, such as anti-inflammation, anti-oxidant and anti-cancer [18,19,20,21]. Resveratrol inhibits the invasion and metastasis of colorectal cancer cells by decreasing nuclear localization of β-catenin [22]. Resveratrol repressed angiogenic activity through the GSK3β (glycogen synthase kinase 3 β/β-catenin/TCF-dependent pathway in human endothelial cells [23]. Recently, Chen *et al.* [24] reported that resveratrol treatment decreased the growth of colon cancer cells and repressed Wnt signaling and expression of its target gene. The proposed mechanism is a disruption of the β-catenin/TCF4 complex without a change in the expression and cellular localization of β-catenin and TCF4.

The aim of the current study is to investigate if TCF4 could be a molecular target of phytochemicals in human colorectal cancer cells. Here, we propose a novel anti-cancer mechanism of resveratrol. Resveratrol increases phosphorylation of TCF4 and decreases the expression of TCF4 through proteasomal degradation in colon cancer cells.

## 2. Results

### 2.1. T-Cell Factor 4 (TCF4) Is a Potential Molecular Target of Phytochemicals in TCF4-Abundant Colorectal Cancer Cells

**Figure 1:**
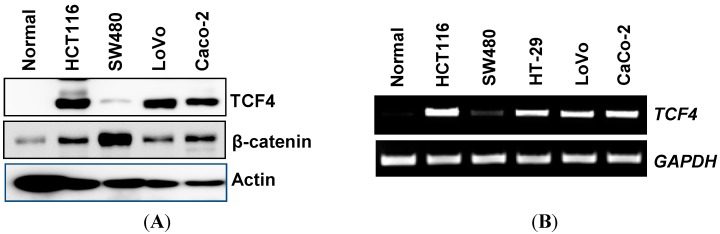

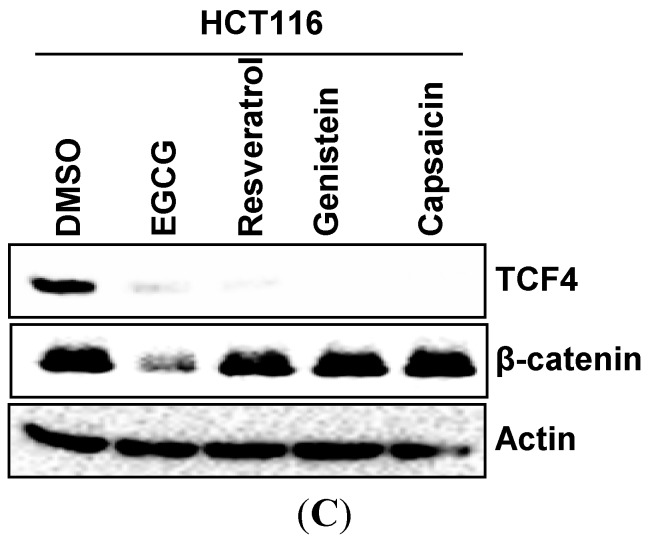
T-cell factor 4 (TCF4) is a potential molecular target of phytochemicals in HCT116 cells. (**A**) Normal human colon cells and different types of human colorectal cancer cells (HCT116, SW480, LoVo and Caco-2) were lysed, and Western blot was performed for TCF4, β-catenin and actin, as described in the Experimental Section; (**B**) Normal human colon cells and different types of human colorectal cancer cells (HCT116, SW480, HT-29, LoVo and Caco-2) were grown overnight. Total RNA was extracted, and RT-PCR was performed using primers for TCF4 and GAPDH; (**C**) HCT116 cells were treated with 50 µM of epigallocatechin gallate (EGCG), resveratrol, genistein and capsaicin for 24 h, and a Western blot was performed for TCF4, β-catenin and actin.

Initially, we selected several types of human colorectal cancer cells with different genetic backgrounds. The genetic information of each type of cell is as follows: HCT116 (APC (adenomatous polyposis coli) wt, β-catenin mut, p53 wt, TGFβR-II (transforming growth factor β receptor-type II) mut, COX-2 (cyclooxygenase-2) null), SW480 (APC del, β-catenin wt, p53 mut, TGFβR-II wt, COX-2 null), HT-29 (APC del, β-catenin wt, p53 mut, TGFβR-II wt, COX-2 wt), LoVo (APC del, β-catenin wt, p53 wt, TGFβR-II mut, COX-2 null) and Caco-2 (APC del, β-catenin mut, p53 null, TGFβR-II mut, COX-2 wt). We performed Western blot and RT-PCR to compare the expression of TCF4 in different human colorectal cancer cells. As shown in Figure 1A, TCF4 was not expressed in normal colon cells, but highly expressed in human colorectal cancer cell lines, including HCT116, LoVo and Caco-2 cells. SW480 cells expressed a lower level of TCF4, which is consistent with other data [25]. Unlikely TCF4, the basal level of β-catenin was high in SW480 cells and relatively lower in other colorectal cancer cell lines. β-catenin expression was relatively low in normal colon cells. The mRNA level of TCF4 showed a similar pattern as protein (Figure 1B). Because HCT116 cells express abundant TCF4 and wild-type adenomatous polyposis coli (APC) gene, we used HCT116 cells for further study.

To test if TCF4 is a target of phytochemicals, we treated the cells with 50 µM of epigallocatechin gallate (EGCG), resveratrol, genistein and capsaicin for 24 h. As shown in Figure 1C, all phytochemicals tested down-regulated TCF4 expression, proposing that down-regulation of TCF4 could be a potential anti-cancer mechanism by phytochemicals in human colorectal cancer. Interestingly, EGCG suppressed expression of TCF4 and β-catenin. We used resveratrol for the further study to investigate the role of TCF4 in cancer prevention.

### 2.2. Resveratrol Down-Regulates TCF4 through Proteasomal Degradation

**Figure 2:**
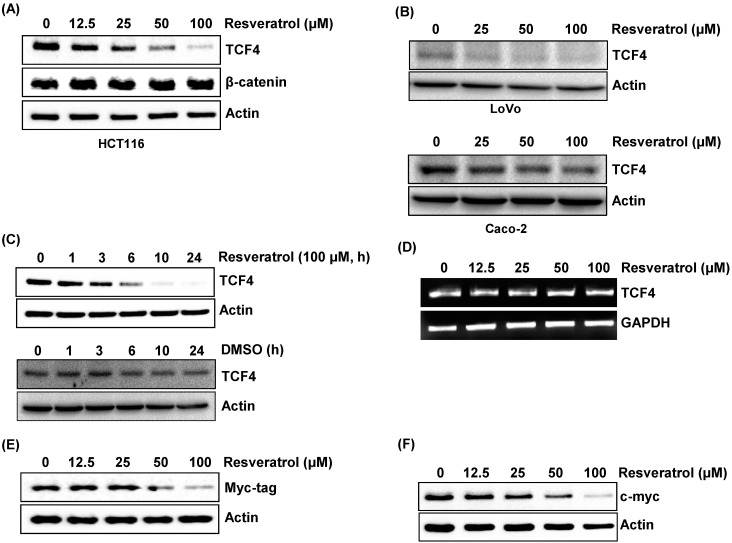
Resveratrol decreases the expression of TCF4 in dose- and time-dependent manner. (**A**) HCT116 cells were treated with indicated concentrations of resveratrol for 24 h in serum-free media, and Western blot was performed for TCF4, β-catenin and actin; (**B**) LoVo and Caco-2 cells were treated with indicated concentrations of resveratrol for 24 h in serum-free media, and Western blot was performed for TCF4 and actin; (**C**) HCT116 cells were treated with 100 µM of resveratrol (upper) or DMSO (lower) for the indicated time points in serum-free media, and Western blot was performed for TCF4 and actin; (**D**) HCT116 cells were treated with indicated concentrations of resveratrol for 24 h in serum-free media; total RNA was isolated, and RT-PCR was performed; (**E**) HCT116 cells were transfected with the myc-tagged TCF4 expression vector and then treated with different concentrations of resveratrol for 24 h, and Western blot was performed using antibody for myc and actin; (**F**) HCT-116 cells were treated with indicated concentrations of resveratrol for 24 h in serum-free media, and Western blot was performed for c-myc and actin.

Next, we observed the effects of resveratrol on TCF4 expression at different doses and time points. The dose of resveratrol was determined based on concentrations to induce apoptosis and cell growth arrest in previous studies [26,27]. As shown in Figure 2A, resveratrol decreased the expression of TCF4 in a dose-dependent manner in HCT116 cells. However, β-catenin expression was not affected by resveratrol treatment. We also tested the effect of resveratrol on TCF4 expression using other human colorectal cancer cells. A marked decrease of TCF4 expression by resveratrol was also observed in LoVo cells, while a slight decrease was shown in Caco-2 cells (Figure 2B). Next, we treated the cells with 100 µM of resveratrol at different time points. TCF4 expression begins to decrease at 6 h after resveratrol treatment (Figure 2C), while it was not changed by treatment with DMSO (vehicle). To observe if a decrease of TCF4 expression is responsible for the transcriptional down-regulation, we tested the mRNA of TCF4 in the cells treated with different doses of resveratrol. As shown in Figure 2D, mRNA was not affected by the treatment with resveratrol. Next, we transfected myc-tagged TCF4 into the cells and then treated with different doses of resveratrol. The result indicates that resveratrol treatment decreased exogenously over-expressed myc-tagged TCF4 protein in a dose-dependent manner (Figure 2E), providing a possibility that resveratrol may decrease the protein stability of TCF4. In addition, resveratrol treatment decreased the expression of the downstream target protein of TCF4, c-myc, in a dose-dependent manner (Figure 2F). This result suggests that TCF4 down-regulation by resveratrol could be enough to down-regulate the β-catenin/TCF4 target gene.

**Figure 3:**
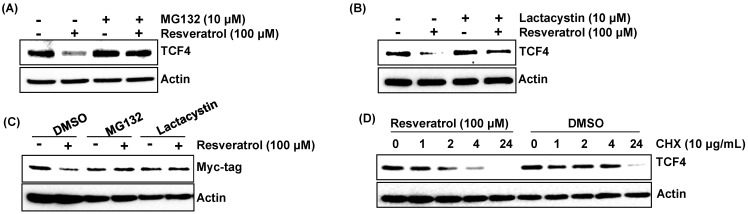
The proteasomal degradation of TCF4 by resveratrol. (**A**,**B**) HCT116 cells were pretreated with different types of proteasomal inhibitors (MG132 (carbobenzoxy-Leu-Leu-leucinal) and lactacystin) for 2 h and then co-treated with resveratrol for 6 h, and Western blot was performed for TCF4 and actin; (**C**) HCT-116 cells were transfected with myc-tagged TCF4 and then pretreated with MG132 and lactacystin for 2 h and then co-treated with resveratrol for 6 h, and Western blot was performed for tag protein (myc) and actin; (**D**) HCT116 cells were pre-treated with DMSO or resveratrol for 2 h and then exposed to cycloheximide (10 µg/mL) for indicated times, and Western blot was performed for TCF4 and actin.

To confirm that resveratrol affects proteasomal degradation of TCF4, the cells were pretreated with two types of proteasome inhibitors (MG132 (carbobenzoxy-Leu-Leu-leucinal) and lactacystin) and then co-treated with resveratrol. As a result, pre-treatment of MG132 and lactacystin blocked resveratrol-induced down-regulation of TCF4 protein (Figure 3A,B). In addition, pretreatment of MG132 and lactacystin ameliorated resveratrol-stimulated degradation of exogenously-introduced myc-tagged TCF4 (Figure 3C). To verify this result, the cells were pre-treated with DMSO or resveratrol and then exposed to cycloheximide for the indicated times. As shown in Figure 3D, resveratrol treatment decreased the half-life of TCF4 protein. Overall, these data proposed that down-regulation of TCF4 by resveratrol depends on proteolytic proteasomal degradation.

### 2.3. Resveratrol Increases TCF4 Phosphorylation

**Figure 4:**
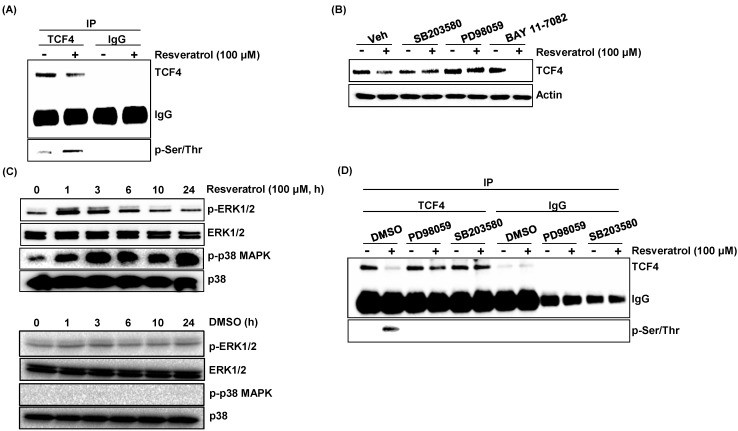
Resveratrol increase phosphorylation of TCF4 in serine/threonine residues through ERK (extracellular signal-regulated kinase) and p38 MAPK (mitogen-activated protein kinase)-dependent pathway. (**A**) HCT116 cells were treated with DMSO or resveratrol for 6 h, and the cell lysates were immunoprecipitated with TCF4 antibody and then immunoblotted with serine/threonine-specific antibody; (**B**) HCT116 cells were pre-treated with selective inhibitors for ERK (PD98059), p38 MAPK (SB203580) and NF-κB (BAY11-7082) for 2 h and then co-treated with resveratrol for 6 h, and Western blot was performed for antibodies against TCF4 and actin; (**C**) HCT116 cells were treated resveratrol (upper) or DMSO (lower) for indicated time points, and then, Western blot was performed for phospho-ERK, ERK, phospho-p38 and p38; (**D**) HCT116 cells were pre-treated with selective inhibitors for ERK (PD98059) and p38 MAPK (SB203580) for 2 h and then co-treated with resveratrol for 6 h, and the cell lysates were immunoprecipitated with TCF4 antibody and then immunoblotted with serine/threonine-specific antibody.

Post-translational modification of TCF4 may affect protein stability and expression of downstream target genes. Several studies have reported on the regulation of TCF4 by phosphorylation [28,29,30]. To investigate whether resveratrol affects TCF4 phosphorylation, the cells were treated with resveratrol, and the cell lysates was pulled down with TCF4 antibody and immunoblotted with phospho-serine/threonine-specific antibody. As a result, resveratrol increased the phosphorylation of TCF4 in serine/threonine residues (Figure 4A).

Because ERK (extracellular signal-regulated kinase) and p38 MAPK (mitogen-activated protein kinase) mediate resveratrol-induced apoptosis [31], we explored if phosphorylation of TCF4 in serine/threonine residues is affected by ERK and p38 MAPK. The cells were pre-treated with selective inhibitors of these kinases and then co-treated with resveratrol. As shown in Figure 4B, pre-treatment of selective inhibitors for p38 (SB203580) and ERK (PD98059) ameliorated resveratrol-induced down-regulation of TCF4. We also tested if resveratrol-stimulated TCF4 down-regulation is mediated by NF-κB, because activation of NF-κB could be an important pro-apoptotic mechanism of chemopreventive agents [32]. The result indicates that TCF4 down-regulation by resveratrol is NF-κB independent.

We also tested if resveratrol induces phosphorylation of ERK and p38 MAPK. As shown in Figure 4C, treatment of resveratrol increased the cellular level of phosphorylated ERK and p38. Next, we performed an immunoprecipitation experiment with the cells pre-treated with inhibitors for ERK and p38 MAPK to observe if these two kinases are responsible for TCF4 phosphorylation. Blocking of these two kinases completely inhibited resveratrol-stimulated phosphorylation of serine and threonine in TCF4. Taken together, resveratrol increased the phosphorylation of TCF4 through ERK and p38 MAPK-mediated pathways.

### 2.4. TCF4 Dependency in Resveratrol Regulation of β-Catenin-dependent Transcriptional Activity

**Figure 5:**
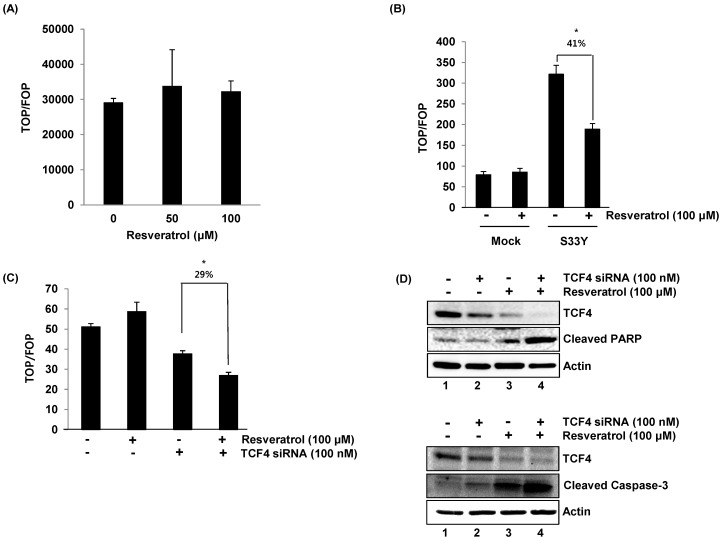
The TCF4 dependency in resveratrol regulation of β-catenin/TCF-dependent transcriptional activity and apoptosis. (**A**) HCT116 cells were transfected with TOPFlash (wild type TCF binding sites) or FOPFlash (mutant TCF binding sites) and then treated with 0, 50 or 100 µM of resveratrol for 24 h. The luciferase activities were measured as described in the Experimental Section; (**B**) HCT116 cells were transfected with empty or pcDNA3-S33Y β-catenin expression vector and then exposed to resveratrol for 24 h; (**C**) HCT116 cells were co-transfected with TOPFlash or FOPFlash and control or TCF4 siRNA. Then, the cells were treated with resveratrol for 24 h; * *p* < 0.05; (**D**) HCT116 cells were transfected with control or TCF4 siRNA and then treated with resveratrol for 24 h. Western blot was performed for antibodies against TCF4, PARP (poly ADP ribose polymerase) and actin.

To investigate whether resveratrol treatment influences the transcriptional activity of β-catenin, we performed a TOPFlash (wild type TCF binding sites) and FOPFlash (mutant TCF binding sites) luciferase reporter assay, which represents β-catenin-dependent TCF transcriptional activity. The results indicate that resveratrol alone did not change the luciferase activity (Figure 5A). Thus, we explored if resveratrol influences β-catenin/TCF-dependent transcriptional activity in a constitutively active condition. The cells were transfected with the S33Y β-catenin mutant. This construct leads to activating β-catenin/TCF-dependent transcriptional activity constitutively, due to a mutation at the phosphorylation site targeted by GSK3β. As a result, there was a 4.1-fold increase of transcriptional activity in the cells overexpressing this constitutively-active β-catenin. However, the treatment of resveratrol suppressed the transcriptional activity by 41% (Figure 5B).

Next, we tested the dependency of TCF4 in β-catenin/TCF-dependent transcriptional activity. The cells were transfected with TCF4 siRNA and treated with resveratrol. As expected, the knockdown of TCF4 suppressed β-catenin/TCF-dependent transcriptional activity in the absence of resveratrol (Figure 5C). Resveratrol did not affect β-catenin/TCF-dependent transcriptional activity in TCF4-expressing cells. However, knockdown of TCF4 leads to enhancing of the resveratrol-stimulated repression of β-catenin/TCF-dependent transcriptional activity by 29%.

To observe that this event affects the cell phenotype, we measured cleaved poly-ADP ribose polymerase (PARP) and caspase-3, which are the hallmark of apoptosis. As shown in Figure 5D, resveratrol treatment minimally increases cleaved PARP (Lane 1 *vs.* 3), but the knockdown of TCF4 dramatically increases resveratrol-stimulated apoptosis (Lane 2 *vs.* 4). Resveratrol also increased cleaved caspase-3 in the cells transfected with TCF4 siRNA. Taken together, our data indicate that TCF4 may sensitize the proapoptotic activity of resveratrol in human colorectal cancer cells.

## 3. Discussion

The Wnt signaling pathway is frequently activated in colorectal cancer. Downstream signaling events are activated through the complex of β-catenin/TCF4, and aberrant regulation of β-catenin leads to progression of colorectal cancer. However, the signaling mechanisms involved and the role of TCF4 in this signal have remained poorly understood. Here, we report that several phytochemicals, including resveratrol, target TCF4, and we propose new mechanisms for which TCF4 may play an important role in phytochemical-induced apoptosis.

We observed that TCF4 and β-catenin were very low in normal cells, indicating that both are oncogenes in colon tissues. The expression patterns of TCF4 and β-catenin were variable in different types of human colorectal cancer cells. It is notable that TCF4 is more abundant in HCT116, LoVo and Caco2 cells than SW480 cells. However, β-catenin expression was much higher in SW480 cells than other colorectal cancer cells.

In our previous study, we observed that capsaicin, a chili pepper component, down-regulated TCF4 without reducing the level of β-catenin protein and subsequently decreased β-catenin/TCF-dependent transcriptional activity in HCT116 adenocarcinoma human colorectal cancer cells, indicating that down-regulation of TCF4 is responsible for β-catenin/TCF-dependent transcriptional activity in capsaicin-treated colorectal cancer cells [16]. Therefore, we tested if other phytochemicals possessing anti-cancer activity negatively affect the expression of TCF4. All phytochemicals tested in this study suppressed expression of TCF4 without changes to β-catenin expression. We also observed that NSAIDs (Non-steroid anti-inflammatory drugs), such as tolfenamic acid, decreased the expression of TCF4 in human colorectal cancer cells (data not shown). These results indicate that the TCF4 could be more likely responsible and a promising target for the suppression of transcriptional activity of β-catenin/TCF-driven gene expression than β-catenin in some types of colon cancers. Therefore, it is reasonable that TCF4 is considered a general target for many anti-cancer compounds.

One of our interesting findings is that resveratrol decreases TCF4 expression through increasing proteasomal degradation. The down-regulation of TCF4 by resveratrol can be important for resveratrol-induced apoptosis and anti-tumorigenesis. One established downstream target of β-catenin/TCF transcription is c-myc, an important cell-cycle regulator. We also found that resveratrol suppressed expression of c-myc, which negatively affects the cell cycle and decreases cell proliferation.

Earlier than our study, Chen *et al.* reported that resveratrol disrupted the binding between β-catenin and TCF4 without changing β-catenin and TCF4 in human colorectal cancer cells [24]. We observed no changes in β-catenin expression, but a decrease in TCF4 expression. We do not know the reason for the inconsistent result for TCF4 expression. We speculate that it might be associated with the different antibody (Millipore, Anti-TCF4, clone 6H5-3, #05-511) used and the treatment method of resveratrol (serum free media).

We also found that down-regulation of TCF4 by resveratrol is dramatic in HCT116 and LoVo cells, but not Caco-2 cells. We do not know why resveratrol down-regulates TCF4 in a cell-type-specific manner in human colorectal cancer cells. It is notable that HCT116 and LoVo cells are p53 wild-type and COX-2 null cells, whereas Caco-2 is p53 null and COX-2 wild-type. Further study will need to elucidate if the status of p53 and COX-2 may affect TCF4 down-regulation by resveratrol.

The current data suggest that phosphorylation of TCF4 by resveratrol might be one of the mechanisms for TCF4 stability, because phosphorylation of TCF4 may affect protein stability and the expression of downstream target genes [28,29,30]. Although we did not identify specific phosphorylation sites in this study, it has been shown that several kinases directly phosphorylate TCF4. For example, Traf2 and Nck-interacting kinase (TNIK) phosphorylates TCF4 on Ser^154^ [28], and homeodomain-interacting protein kinase 2 (HIPK2)-dependent phosphorylation causes the dissociation of TCF4 from its target promoter [30]. TAK1 (transforming growth factor β-activated kinase 1) and MAP (mitogen-activated protein) kinase-related Nemo-like kinase (NLK) phosphorylates TCF on two serine/threonine residues and inhibited DNA binding by the β-catenin/TCF4 complex [33]. However, we do not exclude the possibility that other modifications may affect proteasomal degradation of TCF4. SUMOylation at the Lys35 and Lys297 residues of TCF4 is associated with β-catenin-dependent and TCF4-mediated gene expression [34,35]. The identification of signaling components that are involved in TCF4 phosphorylation and proteasomal degradation in response to phytochemicals will assist in our understanding of Wnt signaling processes coordinating cancer development.

Knockdown of TCF4 efficiently inhibited the TCF-reporter TOPFlash and growth arrest in colon cancer cells [36]. A recent study supports that TCF4 knockdown sensitizes chemotherapeutic agent-mediated cytotoxicity in colon cancer cells [9]. Our data also support that TCF4 knockdown decreased β-catenin/TCF-dependent transcriptional activity and enhanced resveratrol-induced PARP cleavage, which is a hallmark of apoptosis. Because TCF4 has been shown to be constitutively activated by mutated β-catenin and the induction of the pro-apoptotic pathway directly leads to the reduction of TCF4 mRNA and protein levels [37], TCF4 could be a therapeutic target for anti-cancer drugs.

In conclusion, the current study provides information on the molecular events of the anti-cancer activity of resveratrol. Resveratrol increases phosphorylation of TCF4 and proteasomal degradation. Decreased TCF4 represses β-catenin/TCF-dependent transcriptional activity and enhances the proapoptotic activity of resveratrol in human colorectal cancer cells.

## 4. Experimental Section

### 4.1. Reagents

Dulbecco’s modified Eagle medium (DMEM)/F12 1:1 modified medium (DMEM/F12) was purchased from Lonza (Walkersville, MD, USA). Primary antibodies for β-catenin (#9582), myc (#5605), p-ERK (#9101), ERK (#9102), PARP (#9242), cleaved caspase-3 (#9664) and actin (#5125) were purchased from Cell Signaling (Danvers, MA, USA). Antibodies for TCF4 (clone 6H5-3; #05-511) and phospho-serine/threonine (#612548) were purchased from Millipore (Billerica, MA, USA) and BD Transduction Laboratories (San Jose, CA, USA), respectively. Resveratrol, genistein, PD98059, SB203580 and MG-132 were purchased from Calbiochem Biochemicals (San Diego, CA, USA), and epigallocatechin gallate (EGCG), capsaicin and lactacystin were purchased from Sigma-Aldrich (St. Louis, MO, USA). BAY11-7082 was purchased from Enzo Life Science (Farmingdale, NY, USA).

The small interference RNA (siRNA) for the control and TCF4 were purchased from Santa Cruz Biotechnology (Santa Cruz, CA, USA). All chemicals were purchased from Fisher Scientific, unless otherwise specified

### 4.2. Cell Culture and Treatment

Human normal (CCD112CoN) and colorectal cancer cell lines (HCT116, SW480, LoVo and CaCo-2) were purchased from American Type Culture Collection (Manassas, VA, USA) and grown in DMEM/F12 supplemented with 10% fetal bovine serum (FBS), 100 U/mL penicillin and 100 μg/mL streptomycin. The cells were incubated at 37 °C in a humidified atmosphere of 5% CO_2_. All phytochemicals were dissolved in dimethyl sulfoxide (DMSO). DMSO was used as a vehicle, and the final DMSO concentration did not exceed 0.1% (*v*/*v*).

### 4.3. Semi-Quantitative Reverse Transcription-Polymerase Chain Reaction

Total RNA was extracted from the cells using the Qiagen RNeasy Kit (Qiagen, Valencia, CA, USA). One microgram (µg) of total RNA was used for the synthesis of cDNA using the Verso cDNA Synthesis Kit (Thermo Scientific, Pittsburgh, PA, USA) and amplified using the PCR Master Mix (Promega, Madison, WI, USA) according to the manufacturer’s instruction.

The primer sequences for TCF4 are forward 5'-ttcaaagacgacggcgaacag-3' and reverse 5'-ttgctgtacgtgataagaggcg-3'. The primer sequences for GAPDH (glyceraldehyde 3-phosphate dehydrogenase) are forward 5'-gggctgcttttaactctggt-3' and reverse 5'-tggcaggtttttctagacgg-3'. The PCR products were run on 1% agarose gels, stained with ethidium bromide and photographed by a ChemiDoc MP Imaging system (Bio-Rad, Hercules, CA, USA).

### 4.4. Immunoprecipitation and Western Blotting

HCT116 cells were lysed in immunoprecipitation lysis buffer (50 mM Tris–HCl, 150 mM NaCl, 5 mM ethylenediaminetetraacetic acid, 0.5% NP-40) supplemented with protease inhibitor cocktail (Sigma-Aldrich). The cell suspension was centrifuged at 12,000× *g* for 20 min at 4 °C. Protein content was measured by the bicinchoninic acid protein assay (Pierce, Rockford, IL, USA). A total of 500 μg of pre-cleared cell lysates were incubated with anti-TCF4 antibody or normal rabbit immunoglobulin G (Santa Cruz Biotechnology) and Protein A/G PLUS-Agarose (Santa Cruz Biotechnology) overnight with rotation at 4 °C. The pellets were washed five times with lysis buffer and boiled in 2× loading buffer and subjected to Western blot using TCF4 and serine/threonine-specific antibodies.

For immunoblot, equal amounts of proteins were separated by sodium dodecyl sulfate-polyacrylamide gel electrophoresis (SDS-PAGE) and blotted onto nitrocellulose membranes (Osmonics, Minnetonka, MN, USA). After blocking with 5% non-fat dry milk, the membranes were incubated overnight at 4 °C with primary antibodies and then incubated with horse radish peroxidase (HRP)-conjugated immunoglobulin G (IgG) for 1 h at room temperature. Chemiluminescence for protein expression was detected with Pierce ECL Western blotting substrate (Pierce, Rockford, IL, USA) and visualized by the ChemiDoc MP Imaging system (Bio-Rad, Hercules, CA, USA).

### 4.5. Transient Transfection and Enzyme Activity Assay of Luciferase

Transient transfection was performed using PolyJet DNA transfection reagent (SignaGen Laboratories, Ijamsville, MD, USA) according to the manufacturer’s instruction. Briefly, the cells were plated in 12-well plates at the concentration of 2 × 10^5^ cells/well and incubated overnight. Then, plasmid mixtures containing 1 μg of TOPFlash or FOPFlash luciferase plasmid and 0.1 μg of *pRL-null* vector were transfected for 24 h at 37 °C under a humidified atmosphere of 5% CO_2_. The transfected cells were exposed to resveratrol as indicated in the figure legends. Then, the cells were harvested in 1× luciferase lysis buffer, and luciferase activity was measured and normalized as a ratio (*firefly*/*Renilla*), using a dual-luciferase assay kit (Promega, Madison, WI, USA).

### 4.6. Transfection of Small Interference RNA

The cells were transfected with control small interference RNA (siRNA) and TCF4 siRNA for 48 h at a concentration of 100 nM using the TransIT-TKO transfection reagent (Mirus, Madison, WI, USA). Then, the cells were treated with resveratrol for 24 h.

### 4.7. Statistical Analysis

All of the measurements are expressed as the mean ± standard deviation. Statistical analysis was performed with the unpaired Student *t*-test. Differences were considered significant at *p* < 0.05.

## References

[1] Ferlay J., Steliarova-Foucher E., Lortet-Tieulent J., Rosso S., Coebergh J.W., Comber H., Forman D., Bray F. (2013). Cancer incidence and mortality patterns in Europe: Estimates for 40 countries in 2012. Eur. J. Cancer.

[2] Jemal A., Siegel R., Ward E., Hao Y., Xu J., Murray T., Thun M.J. (2008). Cancer Statistics, 2008. CA Cancer J. Clin..

[3] Bos J.L., Fearon E.R., Hamilton S.R., Verlaan-de Vries M., van Boom J.H., van der Eb A.J., Vogelstein B. (1987). Prevalence of *ras* gene mutations in human colorectal cancers. Nature.

[4] Fearon E.R., Hamilton S.R., Vogelstein B. (1987). Clonal analysis of human colorectal tumors. Science.

[5] Fodde R., Smits R., Clevers H. (2001). APC, signal transduction and genetic instability in colorectal cancer. Nat. Rev. Cancer.

[6] Morin P.J., Sparks A.B., Korinek V., Barker N., Clevers H., Vogelstein B., Kinzler K.W. (1997). Activation of β-catenin-TCF signaling in colon cancer by mutations in β-catenin or APC. Science.

[7] Tetsu O., McCormick F. (1999). β-Catenin regulates expression of cyclin D1 in colon carcinoma cells. Nature.

[8] Behrens J., von Kries J.P., Kuhl M., Bruhn L., Wedlich D., Grosschedl R., Birchmeier W. (1996). Functional interaction of β-catenin with the transcription factor LEF-1. Nature.

[9] Xie J., Xiang D.B., Wang H., Zhao C., Chen J., Xiong F., Li T.Y., Wang X.L. (2012). Inhibition of TCF-4 induces apoptosis and enhances chemosensitivity of colon cancer cells. PLoS ONE.

[10] Kim K.J., Lee O.H., Lee B.Y. (2010). Fucoidan, a sulfated polysaccharide, inhibits adipogenesis through the mitogen-activated protein kinase pathway in 3T3-L1 preadipocytes. Life Sci..

[11] Lee S.H., Min K.W., Zhang X., Baek S.J. (2013). 3,3'-diindolylmethane induces activating transcription factor 3 (ATF3) via ATF4 in human colorectal cancer cells. J. Nutr. Biochem..

[12] Jones J.L., Fernandez M.L., McIntosh M.S., Najm W., Calle M.C., Kalynych C., Vukich C., Barona J., Ackermann D., Kim J.E. (2011). A Mediterranean-style low-glycemic-load diet improves variables of metabolic syndrome in women, and addition of a phytochemical-rich medical food enhances benefits on lipoprotein metabolism. J. Clin. Lipidol..

[13] Villa F.A., Gerwick L. (2010). Marine natural product drug discovery: Leads for treatment of inflammation, cancer, infections, and neurological disorders. Immunopharmacol. Immunotoxicol..

[14] COMA (Committee On Medical Aspects) (1998). Nutritional Aspects of the Development of Cancer.

[15] Kundu J.K., Choi K.Y., Surh Y.J. (2006). β-Catenin-mediated signaling: A novel molecular target for chemoprevention with anti-inflammatory substances. Biochim. Biophys. Acta.

[16] Lee S.H., Richardson R.L., Dashwood R.H., Baek S.J. (2012). Capsaicin represses transcriptional activity of β-catenin in human colorectal cancer cells. J. Nutr. Biochem..

[17] Shukla Y., Singh R. (2011). Resveratrol and cellular mechanisms of cancer prevention. Ann. N. Y. Acad. Sci..

[18] Das S., Das D.K. (2007). Anti-inflammatory responses of resveratrol. Inflamm. Allergy Drug Targets.

[19] De la Lastra C.A., Villegas I. (2007). Resveratrol as an antioxidant and pro-oxidant agent: Mechanisms and clinical implications. Biochem. Soc. Trans..

[20] Udenigwe C.C., Ramprasath V.R., Aluko R.E., Jones P.J. (2008). Potential of resveratrol in anticancer and anti-inflammatory therapy. Nutr. Rev..

[21] Trincheri N.F., Nicotra G., Follo C., Castino R., Isidoro C. (2007). Resveratrol induces cell death in colorectal cancer cells by a novel pathway involving lysosomal cathepsin D. Carcinogenesis.

[22] Ji Q., Liu X., Fu X., Zhang L., Sui H., Zhou L., Sun J., Cai J., Qin J., Ren J. (2013). Resveratrol inhibits invasion and metastasis of colorectal cancer cells via MALAT1 mediated Wnt/β-catenin signal pathway. PLoS ONE.

[23] Wang H., Zhou H., Zou Y., Liu Q., Guo C., Gao G., Shao C., Gong Y. (2010). Resveratrol modulates angiogenesis through the GSK3β/β-catenin/TCF-dependent pathway in human endothelial cells. Biochem. Pharmacol..

[24] Chen H.J., Hsu L.S., Shia Y.T., Lin M.W., Lin C.M. (2012). The β-catenin/TCF complex as a novel target of resveratrol in the Wnt/β-catenin signaling pathway. Biochem. Pharmacol..

[25] Korinek V., Barker N., Morin P.J., van Wichen D., de Weger R., Kinzler K.W., Vogelstein B., Clevers H. (1997). Constitutive transcriptional activation by a β-catenin-TCF complex in APC^−/−^ colon carcinoma. Science.

[26] Miki H., Uehara N., Kimura A., Sasaki T., Yuri T., Yoshizawa K., Tsubura A. (2012). Resveratrol induces apoptosis via ROS-triggered autophagy in human colon cancer cells. Int. J. Oncol..

[27] Fouad M.A., Agha A.M., Merzabani M.M., Shouman S.A. (2013). Resveratrol inhibits proliferation, angiogenesis and induces apoptosis in colon cancer cells: Calorie restriction is the force to the cytotoxicity. Hum. Exp. Toxicol..

[28] Shitashige M., Satow R., Jigami T., Aoki K., Honda K., Shibata T., Ono M., Hirohashi S., Yamada T. (2010). Traf2- and Nck-interacting kinase is essential for Wnt signaling and colorectal cancer growth. Cancer Res..

[29] Sokol S.Y. (2011). Wnt signaling through T-cell factor phosphorylation. Cell Res..

[30] Hikasa H., Sokol S.Y. (2011). Phosphorylation of TCF proteins by homeodomain-interacting protein kinase 2. J. Biol. Chem..

[31] She Q.B., Bode A.M., Ma W.Y., Chen N.Y., Dong Z. (2001). Resveratrol-induced activation of p53 and apoptosis is mediated by extracellular-signal-regulated protein kinases and p38 kinase. Cancer Res..

[32] Jeong J.B., Yang X., Clark R., Choi J., Baek S.J., Lee S.H. (2013). A mechanistic study of the proapoptotic effect of tolfenamic acid; involvement of NF-κB activation. Carcinogenesis.

[33] Ishitani T., Ninomiya-Tsuji J., Matsumoto K. (2003). Regulation of lymphoid enhancer factor 1/T-cell factor by mitogen-activated protein kinase-related Nemo-like kinase-dependent phosphorylation in Wnt/β-catenin signaling. Mol. Cell. Biol..

[34] Ihara M., Yamamoto H., Kikuchi A. (2005). SUMO-1 modification of PIASy, an E3 ligase, is necessary for PIASy-dependent activation of TCF-4. Mol. Cell. Biol..

[35] Yamamoto H., Ihara M., Matsuura Y., Kikuchi A. (2003). Sumoylation is involved in β-catenin-dependent activation of TCF-4. EMBO J..

[36] Van de Wetering M., Sancho E., Verweij C., de Lau W., Oving I., Hurlstone A., van der Horn K., Batlle E., Coudreuse D., Haramis A.P. (2002). The β-catenin/TCF-4 complex imposes a crypt progenitor phenotype on colorectal cancer cells. Cell.

[37] Rother K., Johne C., Spiesbach K., Haugwitz U., Tschop K., Wasner M., Klein-Hitpass L., Moroy T., Mossner J., Engeland K. (2004). Identification of TCF-4 as a transcriptional target of p53 signalling. Oncogene.

